# Neuromechanics of finger hangs with arm lock-offs: analyzing joint moments and muscle activations to improve practice guidelines for climbing

**DOI:** 10.3389/fspor.2023.1251089

**Published:** 2023-10-20

**Authors:** Juliana Exel, David Deimel, Willi Koller, Cäcilia Werle, Arnold Baca, Daniela Maffiodo, Raffaella Sesana, Alessandro Colombo, Hans Kainz

**Affiliations:** ^1^ Department of Biomechanics, Kinesiology and Computer Science in Sport, Centre for Sport Science and University Sports, University of Vienna, Vienna, Austria; ^2^ Neuromechanics Research Group, Centre for Sport Science and University Sports, University of Vienna, Vienna, Austria; ^3^ Vienna Doctoral School of Pharmaceutical, Nutritional and Sport Sciences, University of Vienna, Vienna, Austria; ^4^ Department of Mechanical and Aerospace Engineering, Politecnico di Torino, Turin, Italy; ^5^ Department of Electronics, Information and Bioengineering, Politecnico di Milano, Milan, Italy

**Keywords:** climbing, neuromechanics, arm lock-offs, injury prevention, training optimization

## Abstract

**Introduction:**

Climbing imposes substantial demands on the upper limbs and understanding the mechanical loads experienced by the joints during climbing movements is crucial for injury prevention and optimizing training protocols. This study aimed to quantify and compare upper limb joint loads and muscle activations during isometric finger hanging exercises with different arm lock-off positions.

**Methods:**

Seventeen recreational climbers performed six finger dead hangs with arm lock-offs at 90° and 135° of elbow flexion, as well as arms fully extended. Upper limb joint moments were calculated using personalized models in OpenSim, based on three-dimensional motion capture data and forces measured on an instrumented hang board. Muscle activations of upper limb muscles were recorded with surface electromyography electrodes.

**Results:**

Results revealed that the shoulder exhibited higher flexion moments during arm lock-offs at 90° compared to full extension (*p* = 0.006). The adduction moment was higher at 135° and 90° compared to full extension (*p* < 0.001), as well as the rotation moments (*p* < 0.001). The elbows exhibited increasing flexion moments with the increase in the arm lock-off angle (*p* < 0.001). Muscle activations varied across conditions for biceps brachii (*p* < 0.001), trapezius (*p* < 0.001), and latissimus dorsi, except for the finger flexors (*p* = 0.15).

**Discussion:**

Our findings indicate that isometric finger dead hangs with arms fully extended are effective for training forearm force capacities while minimizing stress on the elbow and shoulder joints. These findings have important implications for injury prevention and optimizing training strategies in climbing.

## 1. Introduction

The importance of sustained isometric strength in the fingers and forearm muscles for climbing success has been well-established (1). Climbers face the challenge of harnessing this specific strength capacity to navigate a large variety of climbing styles, characterized by different hold shapes, orientations, and wall steepness. Consequently, the upper limbs have consistently been identified as the most vulnerable to injuries across all levels of performance, age, and gender (2–5), with injuries due to overuse being particularly prevalent (6, 7). Notably, a significant proportion (42% to 71%) of climbing injuries occur in the wrists, elbows, and shoulders, resulting from overuse or acute atraumatic incidents (2).

The mechanical loading experienced by the body can lead to physiological adaptations and therefore impact performance and function of the musculoskeletal system (1, 8). These loading patterns, characterized by interacting physical forces—magnitude, duration, frequency, rate of force development, type, and direction of application—yield various effects on the tissues, ranging from favorable functional adaptations (e.g., increased strength, coordinated movement) to potential chronic overload injuries (9). Previous studies investigating mechanical loading in climbing have predominantly focused on the fingers, either *in vivo* or *in situ*. These studies examined finger force capacities under different hold depths and grip techniques and highlighted that maximal forces increase with the hold depth, with crimping requiring higher finger flexion force (10–12). Biomechanical models have been applied to estimate the forces acting on finger tendons and pulleys during specific climbing grip techniques and indicated that crimping elicits higher forces on the finger pulleys compared to more open grip techniques (13), while also demanding greater forces on the ring and middle fingers (14). To the best of the authors' knowledge, no studies evaluated the mechanical loads during climbing or climbing-related activities at other anatomical structures, e.g., elbow and shoulder joints. Considering that these joints are prone to injuries (2), quantifying elbow and shoulder loads might help to enhance our understanding of certain injury mechanisms and prevent overuse injuries in the future.

One aspect associated with climbing performance is the lock-off ability (15). This term refers to the gesture involved in pulling movements during ascent. While climbers apply force with one hand to the initiating hold (H), they release the other hand to reach the next target hold (T). During this brief period, known as lock-off, H engages in static and isometric exertion. Once the target hold is reached, H and T often remain in a partially locked-off state, enabling climbers to regain balance and execute the necessary footwork for the subsequent move (16). Lock-offs are performed across a range of upper-body joint angles, depending on the steepness of the climbing surface and the initial and final positions between subsequent holds (16). In some instances, climbers can utilize lower limb support to perform the movement, while in others, they cannot. Consequently, the intensity of a lock-off also depends on the type of movement being executed during the ascent. Under such conditions, climbers may experience varying degrees of joint loading.

Gaining a better understanding of the neuromechanical behavior during climbing movements could enhance the quality of training protocols by ensuring effectiveness and mitigating injury risks. Therefore, the aim of the present study was to quantify and compare upper limb joint loads and muscle activations between three isometric finger hanging exercises with specific lock-off positions, i.e., (1) elbows flexed at 90°, (2) elbows flexed at 135°, and (3) elbows fully extended at 180°. We hypothesized that shoulder and elbow joint loads will increase with increasing elbow flexion, whereas forearm muscle activations will remain the same.

## 2. Methods

### 2.1. Sample

A total of 17 recreational climbers (age: 26.3 ± 3.7; height: 1.70 ± 0.1 m; weight: 62.0 ± 9.2 kg) were recruited to participate in the study. The sample consisted of advanced/elite climbers, with a mean ability rating of 22.1 ± 1.8 according to the IRCRA reporting scale (17). To be eligible for participation, climbers had to have prior experience using a hang board, which is a commonly used instrument for finger strength training. Additionally, participants were required to have no history of upper-limb musculoskeletal injuries that could hinder their involvement in the study. The research protocol received approval from the Ethics Committee of the University of Vienna (00690), and all participants were provided with detailed information about the study's objectives. Before participating, they voluntarily signed an informed consent form indicating their willingness to take part in the research.

### 2.2. Experimental protocol

**Figure 1:**
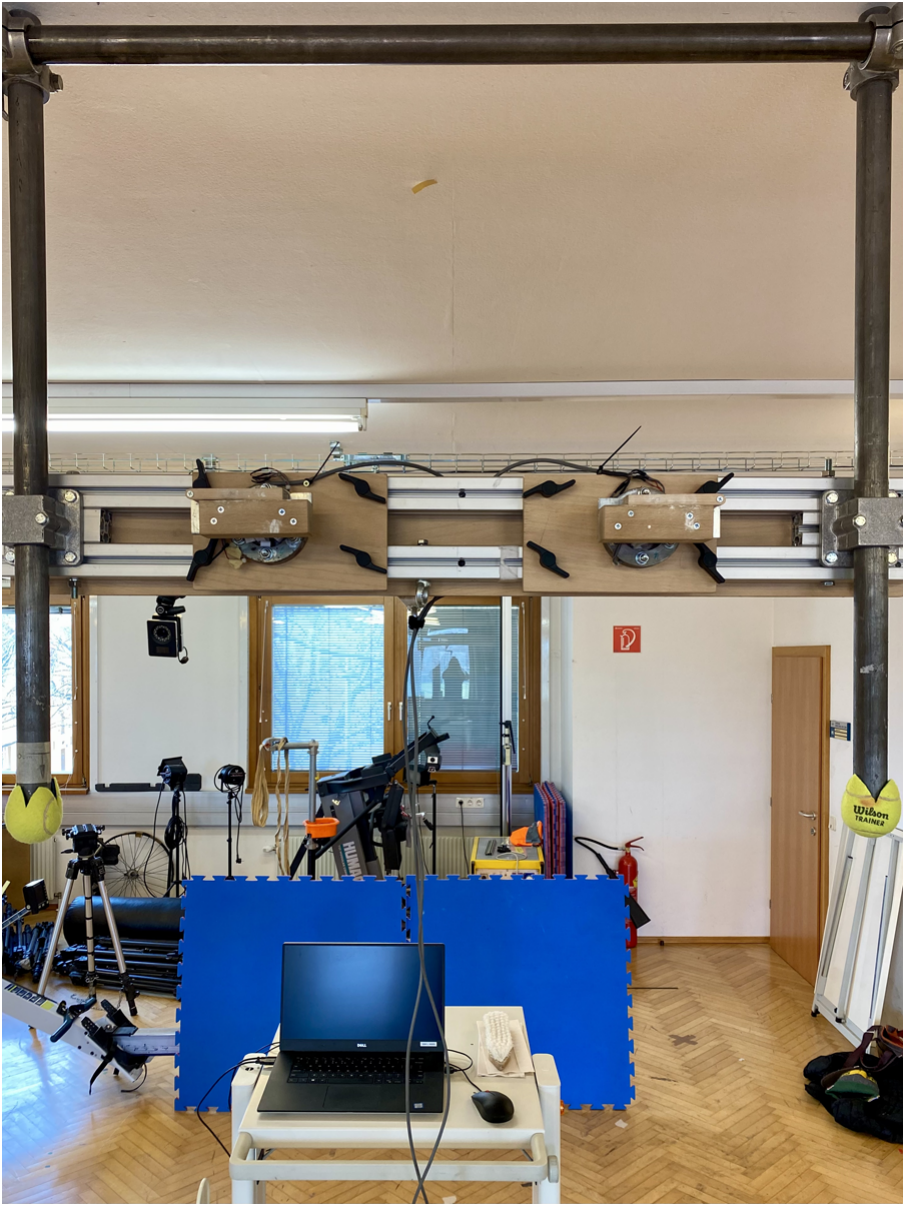
Instrumented hang board used in the study. The force sensors were placed in separate hand holds, with height and width being adjusted according to participant's individual anthropometry and the desired arm lock-off angles required in the tasks.

**Figure 2:**
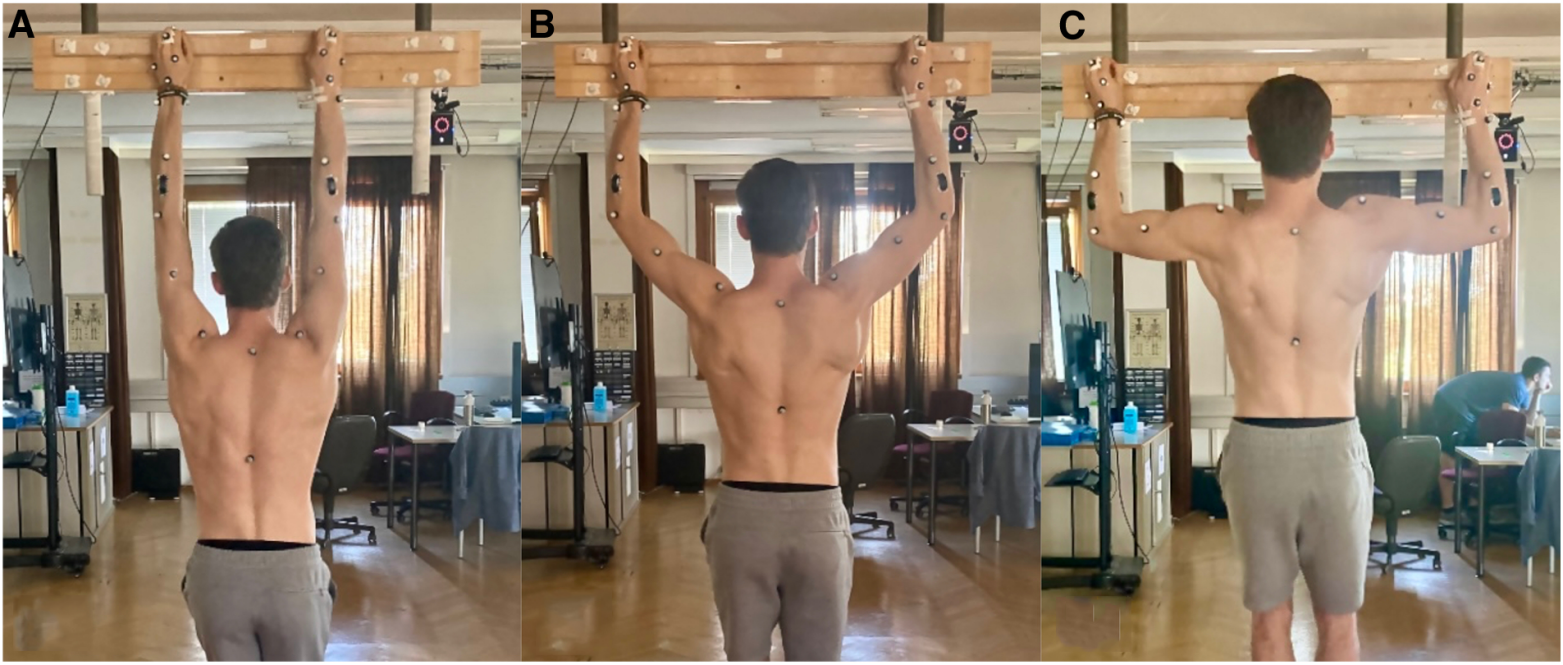
Dead hang exercises performed in the present study. (**A**) represents the hanging performed at full elbow extension (180°). (**B**) indicates the arm lock-off at 135° of elbow flexion, and in (**C**) it is represented the position of the participants during the lock-off at 90° of elbow flexion.

The experimental protocol involved conducting six trials of isometric finger hangings on a custom-designed and instrumented hang board consisted of 2 separate handles, and utilizing a 22 mm-depth edge (Figure 1). The hangings were performed with fingers positioned in an open crimp grip, under three different conditions: elbows flexed at 90° (Figure 2C), 135° (Figure 2B), and in full 180° extension (Figure 2A), and the order was self-selected by the participant. The distance between the handles was adjusted to enable participants to perform the task with the desired elbow flexion positions, confirmed with the help of a goniometer, while maintaining the forearm vertical. Each position was held for a duration of 12 s. A one-minute rest period was provided between trials, while a five-minute rest period was given between conditions. With this design, the low-intensity exercises could be carried out by the participants without interference from previous training or climbing sessions. Prior to commencing data collection, participants were instructed to engage in a 10-min warm-up routine. They were allowed to choose between a self-selected routine or a suggested routine, which included joint-mobility exercises, rowing, push-ups, and assisted finger dead hangs.

### 2.3. Measurements

**Figure 3:**
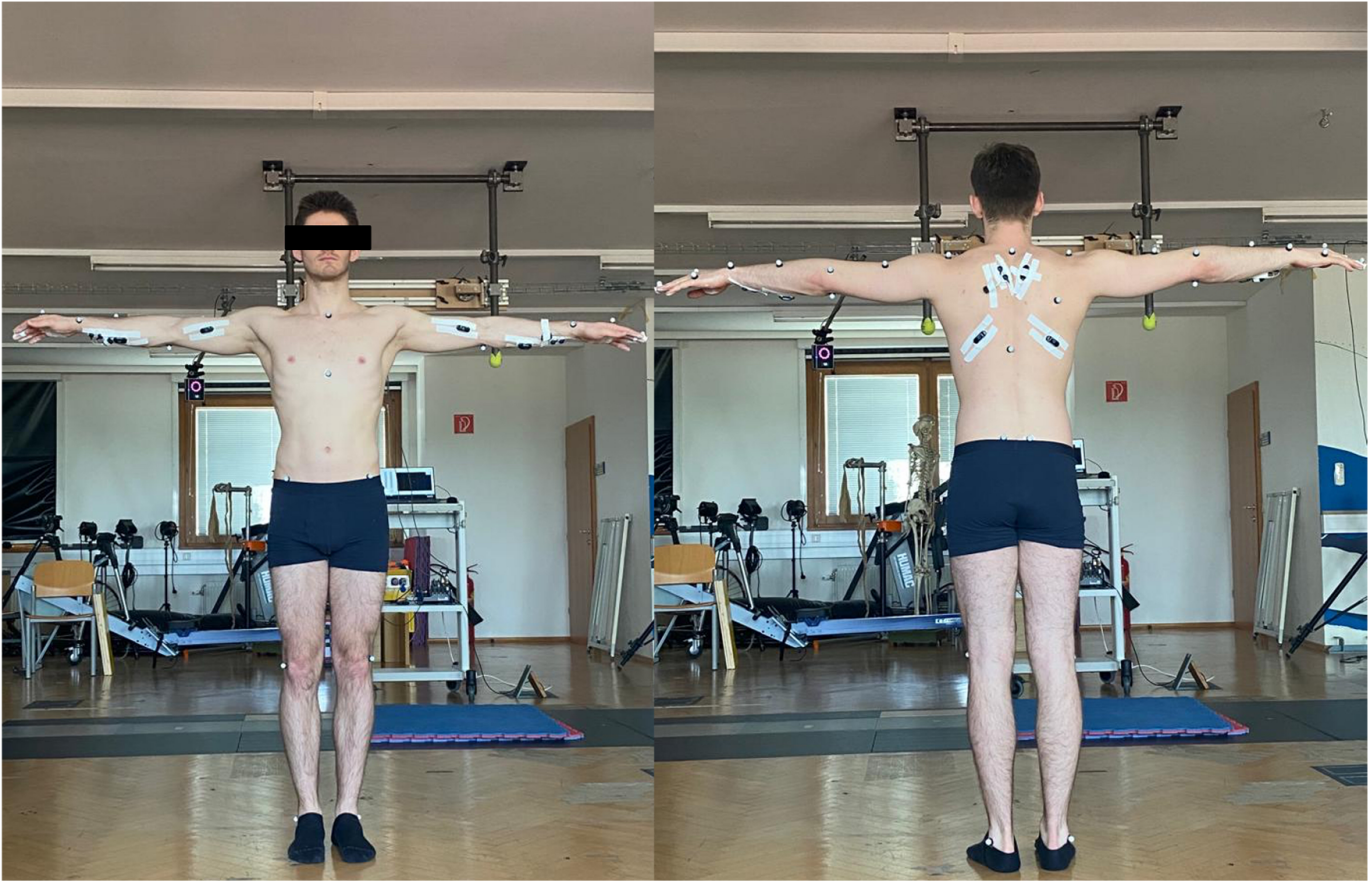
Participant with surface markers and EMG sensors. The leg markers are not included in the Plug-in-Gait model used in this study for the kinematic measurements, but it was applied for visualization purposes only.

To capture the body kinematics of our participants, a total of 33 retroreflective surface markers were attached to each participant (Figure 3), and their trajectories were recorded using a 12-camera motion capture system (Vicon Motion Systems, Oxford, UK) at a sampling frequency of 200 Hz. The marker model used was a modified version of the Plug-in-Gait marker set (18), with additional markers placed on the phalanx distalis, as well as the index and pinky fingers. Following data collection, the markers were labeled, gap-filled, and low-pass filtered using Nexus 2.14.0 software (Vicon Motion Systems, Oxford, UK).

Electromyographic signals (EMG) from the finger digitorium superficialis (finger flexor), biceps brachii—long head (biceps brachii), trapezius, and latissimus dorsi were recorded from both left and right limbs using a wireless system (Cometa®, Milan, Italy) at a sampling rate of 2,000 Hz, synchronized with the motion capture system. The placement of surface electrodes followed the SENIAM guidelines (19) for all muscles, except for the finger flexor, which was placed according to Vigouroux et al. (20). The recorded EMG data was filtered using an 4th-order band-pass filter with cutoff frequencies of 6 Hz and 600 Hz, and demeaned (21).

Forces applied during the finger hangings in the vertical and medial-lateral directions were measured using force sensors mounted on the hang board (Figure 1). These 2D sensors are based on 4 HBM strain gauges for each direction, as Wheatstone bridge circuit, mounted on a National Instruments cDAQ-9174. For further details, see Maffiodo et al. (22). The force sensor was synchronized with the motion capture system and collected data at a sampling frequency of 1,000 Hz.

### 2.4. Data processing

#### 2.4.1. Estimation of joint loads

OpenSim (23) was used to quantify wrist, elbow and shoulder angles and moments for each trial. The Rajagopal model (24) was slightly modified to ensure an adequate range of motion in the upper limb joints for the tasks performed. In this model, the shoulder joint was represented as a ball and socket joint with three degrees-of-freedom (DoF), while the elbow and wrist joints included two DoF, enabling flexion/extension and pronation/supination at the elbow, and radial/ulnar deviations at the wrist.

To personalize the model, the generic model was scaled based on the surface marker locations from a static trial to match each participant's anthropometry. This is performed by comparing the experimental marker data from the motion capture to the virtual markers from the Rajagopal model used. Subsequently, the personalized model and the corresponding marker trajectories from the arm lock-offs were used to calculate joint angles using inverse kinematics. The vertical and lateral forces measured with the force sensors were applied to the hand segment of the model at the location of the finger markers. Inverse dynamics analysis was employed to compute joint moments for all degrees of freedom. The joint moments were then smoothed using a LOESS function, and the parameters were defined after residual analysis and inspection of the derivatives. The peak values from the middle 10 ms of each trial were extracted. Additionally, peak joint moments were normalized by participant's body weight for further analysis. Therefore, the upper limb loads were normalized by individual's body weight (Nm/kg) and are represented by their estimated peak joint moments, defined as follows: flexion (+) and extension (−) in the sagittal plane; internal rotation/pronation (+) and external rotation/supination (−) in the transverse plane; adduction/radial deviation (+) and abduction/ulnar deviation (−) in the frontal plane.

#### 2.4.2. Estimation of muscle activity

Muscle activity in the upper body was assessed using the root mean square (RMS) of the recorded EMG signals from the finger flexor, biceps brachii, trapezius, and latissimus dorsi muscles. The RMS was computed with a window size of 250 ms and overlaps of 125 ms. Data was amplitude-normalized by the peak activation observed in the trials performed at 180° elbow condition. For analysis purposes, the peak RMS-relative to 180° values from the middle windows of each trial were expressed as a percentage and will be presented accordingly (%RMS180°).

### 2.5. Statistical analysis

Prior to the analyses, data normality was assessed using the Shapiro-Wilk test. For normally distributed data, comparisons were performed using ANOVA for repeated measures with Bonferroni correction for multiple comparison, and the results were reported accordingly. In the case of non-normally distributed data, Friedman's Two-way Analysis of Variance by Ranks Summary was applied. Side differences were tested using the Wilcoxon signed rank test. In all tests, statistical significance was considered when *p* < 0.05. Data analysis was carried out using custom-built scripts in MATLAB 2022a (MathWorks Inc., Natick, MA, USA) and IBM SPSS Statistics 29.0.0.0 (Armonk, NY: IBM Corp).

## 3. Results

**Figure 4:**
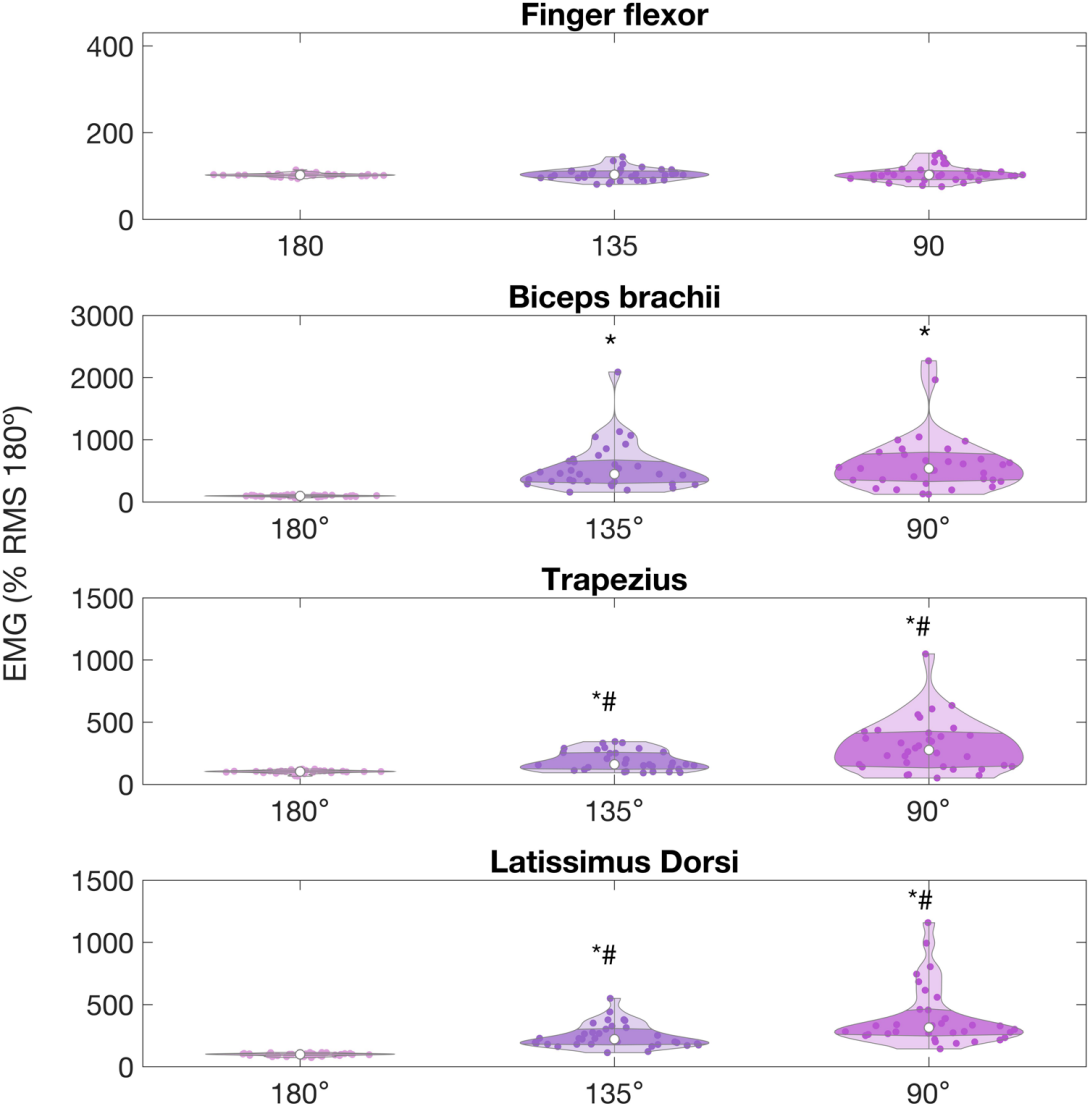
Average EMG RMS, represented as % relative to the peak value observed at 180°, for upper limb muscles of climbers during arm lock-offs performed at full elbow extension (180°), 135° and 90° of elbow flexion, in a 22-mm depth ledge. Values are expressed as percentage of the peak normalized by the full elbow extension condition. (*) indicates significant difference with the 180° condition. (^#^) indicates significant difference with 135° condition.

Friedman's showed that the muscle activation obtained for all of the upper body muscles across the arm lock-off conditions was significantly different for all muscles (*χ*^2^(2) = 52.62, *p* < 0.001 for the biceps brachii; *χ*^2^(2) = 52.51, *p* < 0.001 for the trapezius; *χ*^2^(2) = 62.90, *p* < 0.001 for the latissimus dorsi) except for the finger flexors (*χ*^2^(2) = 1.55, *p* = 0.45). We found significantly higher %RMS180° at 135° and 90° when compared to full elbow extension, and no differences were found between 135° and 90°, as can be seen in in Figure 4.

The mean %RMS180° (± standard deviation) recorded for the finger flexor was 102.2 ± 33.2%, 103.4 ± 14.2%, and 102.8 ± 18.42% at full extension, 135°, and 9°, respectively, and did not change across conditions. Biceps brachii %RMS180° was significantly lower when participants performed at full extension (97.2% ± 12.0%), compared to the arm lock-off at 135° (447.26 ± 386.21%, *p* < 0.001) and 90° (524.5% ± 468.5, *p* < 0.001). No differences were found when comparing biceps brachii at 90° and 135° (*p* = 0.90). Trapezius and latissimus dorsi %RMS180° were lower at full extension (103.02 ± 13.00%, and 99.67 ± 10.52%, respectively) when compared to 135° (158.42 ± 90.02%, 212.03 ± 104.65%, respectively, with *p* = 0.001), and 90° (277.67 ± 207.09%, and 314.74 ± 236.00%, *p* < 0.001). Both muscles also showed significant differences when compared between 135° and 90° (*p* = 0.002 and *p* = 0.001, respectively).

The ANOVA showed that the joint moments differed between arm lock-offs at different conditions (Table 1). The shoulder presented significant differences in the moments in all planes [F (1,33) = 23.54, *p* < 0.001]. The adduction moment was higher [F (1,33) = 93.80, *p* < 0.001] for the conditions at 135° and 90° when compared to arms fully extended (*p* < 0.001 for both comparisons). The internal-external rotation moments at the shoulder were significantly different across all conditions [F (1,33) = 471.41, *p* < 0.001], being higher in the lock-offs performed at 90° and at 135° compared to arms at full extension (*p* < 0.001 in all comparisons).

**Table 1:** Estimated external upper body joint moments of climbers during dead hang exercises performed with arm lock-offs at different degrees of elbow flexion.

Joint Moments (Nm/kg)							
Arm lock-off condition	Shoulder sagittal	Shoulder frontal	Shoulder transversal	Elbow sagittal	Elbow transversal	Wrist sagittal	Wrist frontal
90°	0.56 ± 0.18*^,^^#^	0.53 ± 0.22*	0.41 ± 0.10*^,^^#^	0.39 ± 0.16*^,^^#^	0.001 ± 0.03*	0.30 ± 0.04	−0.02 ± 0.06*
135°	0.49 ± 0.16	0.45 ± 0.16*	0.23 ± 0.07*	0.24 ± 0.14*	0.02 ± 0.02^#^	0.31 ± 0.05*	−0.002 ± 0.062*
Full elbow extension	0.41 ± 0.15	0.19 ± 0.09	0.07 ± 0.03	0.13 ± 0.07	0.01 ± 0.01	0.30 ± 0.04	0.07 ± 0.05
*p*-value*	**0** **.** **006**	**<0** **.** **001**	**<0** **.** **001**	**<0** **.** **001**	**0** **.** **02**	**1** **.** **00**	**<0** **.** **001**
*p*-value^#^	**0** **.** **005**	**<0** **.** **001**	**<0** **.** **001**	**<0** **.** **001**	**<0** **.** **001**	**0** **.** **42**	**0** **.** **13**
*p*-value^ø^	**0** **.** **006**	**0** **.** **08**	**<0** **.** **001**	**<0** **.** **001**	**0** **.** **81**	**0** **.** **02**	**<0** **.** **001**

*p*-value* of the comparison between 90°–180°; *p*-value^#^ of the comparison between 90°–13°; *p*-value^ø^ of the comparison between 180°–135°.

* Significantly different from full elbow extension.

^#^ Significantly different from 135°.

^ø^ Significantly different from 90°. Movements in the sagittal plane: flexion/extension (+/−); Movements in the transversal plane: internal—pronation/external—supination rotations (+/−); Movements in the frontal plane: adduction—ulnar deviation/ abduction—radial deviation (+/−).

For the elbow in the sagittal plane, ANOVA also revealed that the external joint moments were different across all exercise conditions [F (1,33) = 88.77, *p* < 0.001]. The highest moments were found when participants performed arm lock-offs at 90°, followed by lock-offs at 135°, when compared with arms fully extended. Although the magnitudes were considerably small, pronation moments were significantly lower in 90° lock-offs when compared to 180° and also lower for 135° when compared to 90° [F (1,33) = 8.55, *p* = 0.006].

The ANOVA showed that the wrist did not present significant differences in the sagittal plane [F (1,33) = 0.85, *p* = 0.36] but did for the movements in the frontal [F (1,33) = 35.46, *p* < 0.001]. When participants performed the exercises with elbows fully extended, the moments significantly changed from a small radial deviation to ulnar deviation moment as the degree of arm lock-offs decreased from 90° and 135° to arms fully extended.

**Figure 5:**
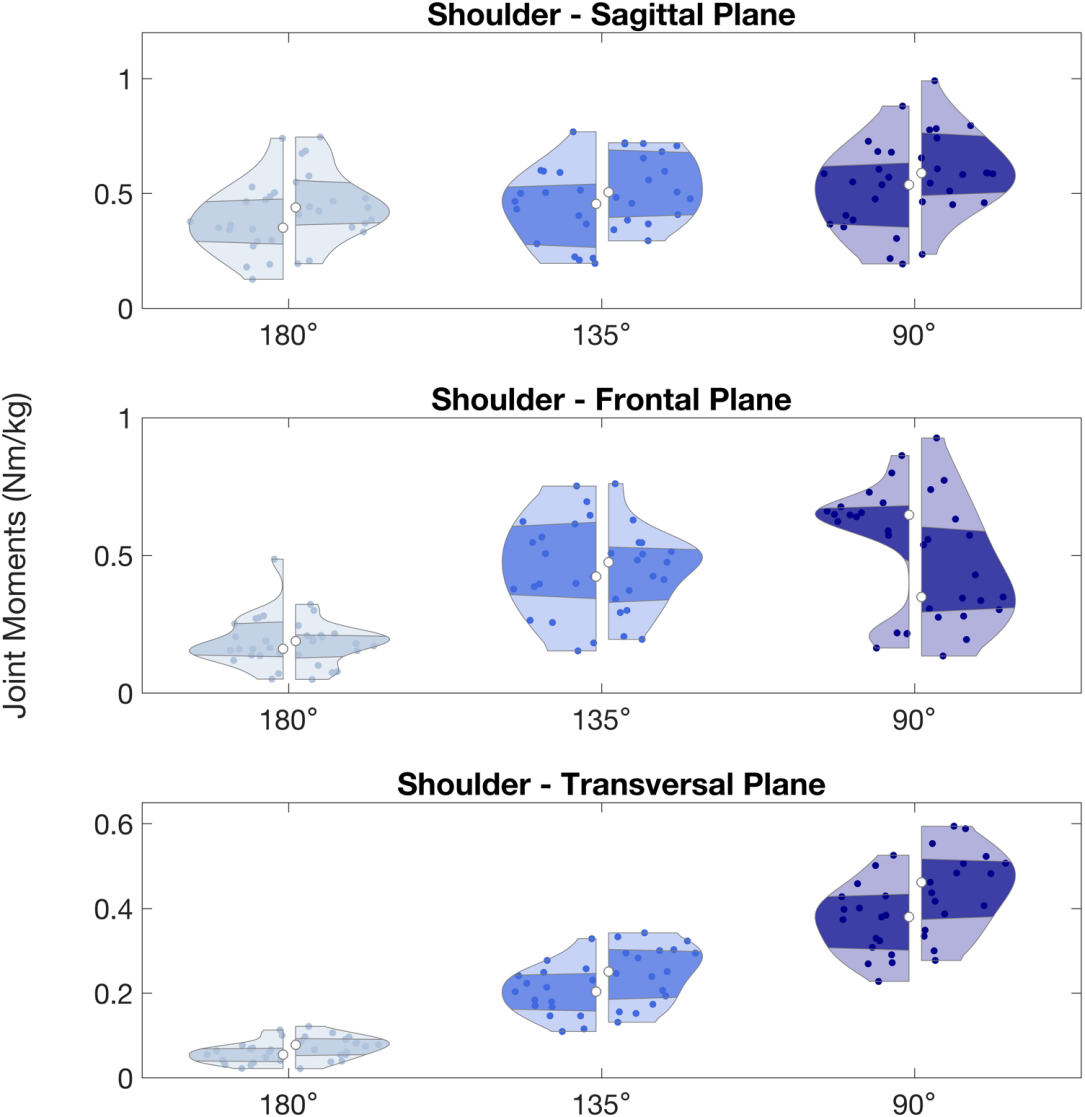
Shoulder peak joint moments (Nm/kg) observed during arm lock-offs performed by climbers at full elbow extension (180°), 135° and 90° of elbow flexion. Left and right sides of the violin plots represent the left and right upper limbs. (*) indicates significant difference between left and right sides.

**Figure 6:**
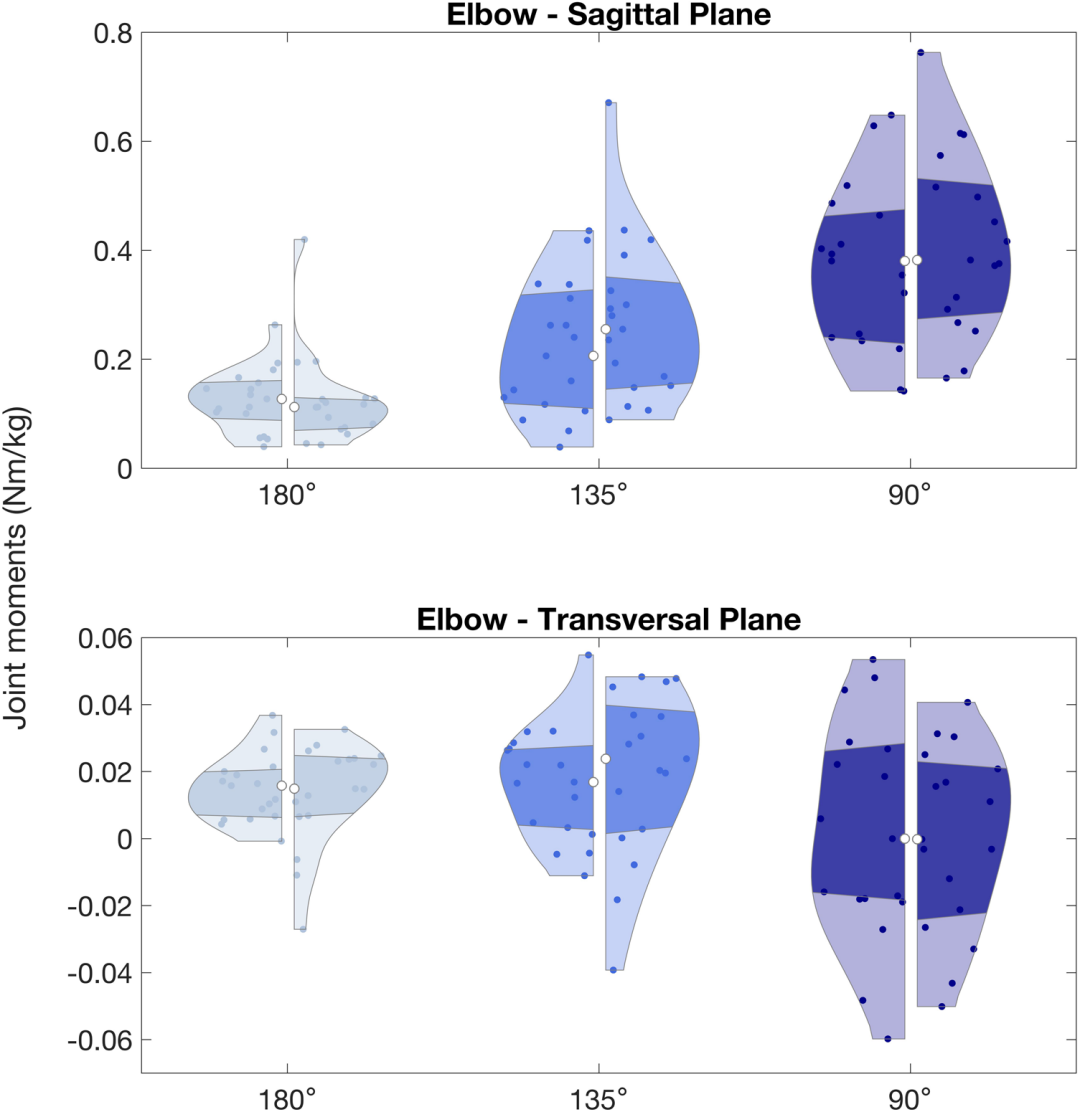
Elbow peak joint moments (Nm/kg) observed during arm lock-offs performed by climbers at full elbow extension (180°), 135° and 90° of elbow flexion. Left and right sides of the violin plots represent the left and right upper limbs.

**Figure 7:**
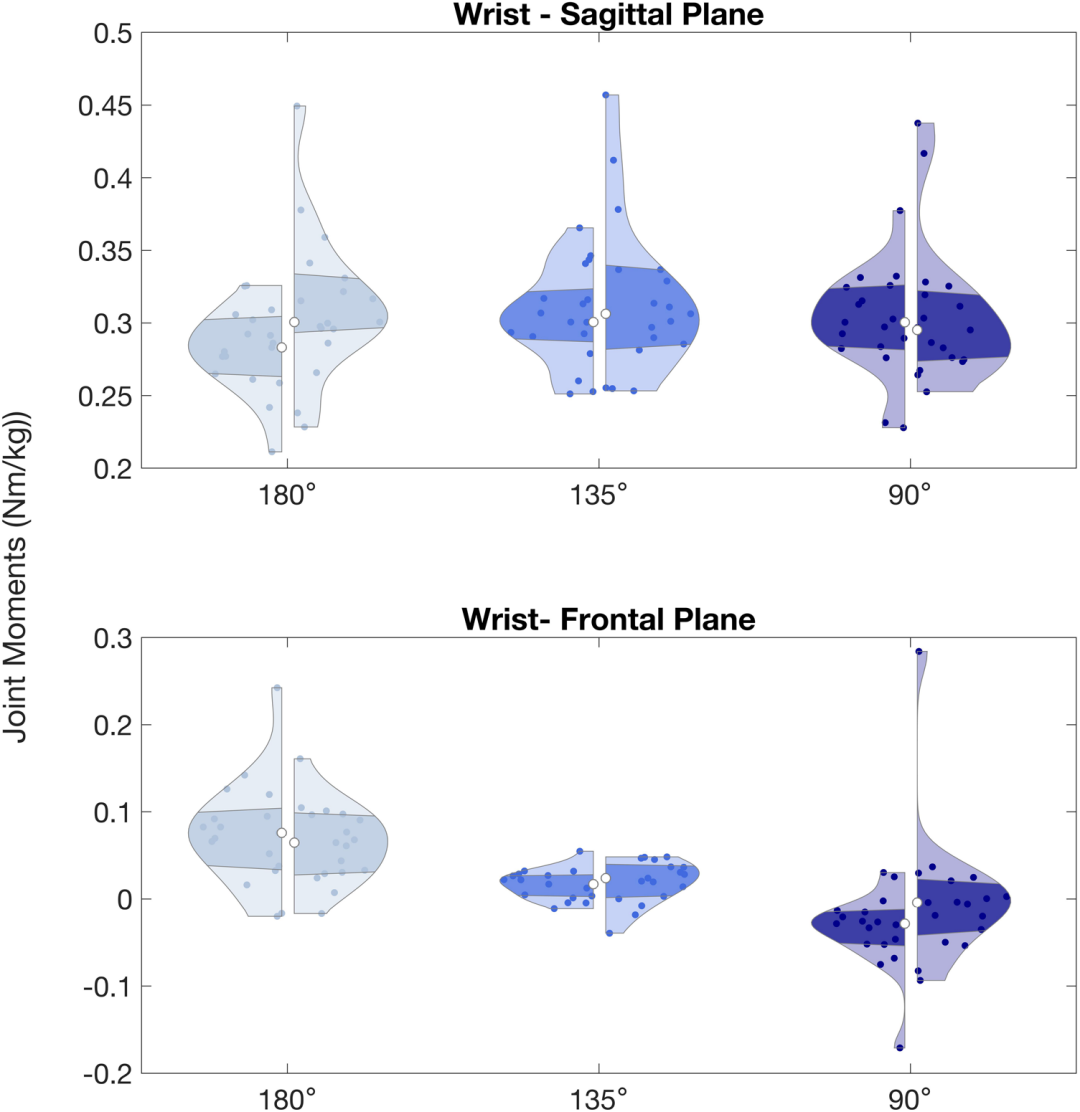
Wrist peak joint moments (Nm/kg) observed during arm lock-offs performed by climbers at full elbow extension (180°), 135° and 90° of elbow flexion. Left and right sides of the violin plots represent the left and right upper limbs. (*) indicates significant difference between left and right sides.

Wilcoxon results on the side imbalances for some joints showed significant differences across arm lock-off conditions, and seemed to increase concomitantly with increasing elbow flexion angles, as can be seen in Figures 5–7. The shoulder moments in the sagittal plane presented left-right significant differences at arms fully extended (absolute differences ± standard deviation: −0.08 ± 0.09 Nm/kg, Z = −2.91, *p* = 0.003), at 135° (−0.10 ± 0.15 Nm/kg; Z = −2.15, *p* = 0.03), but not at 90° (0.11 ± 0.25 Nm/kg; Z = −1.77, *p* = 0.07). The shoulder in the transversal plane also had left-right significant differences in all conditions (−0.02 ± 0.02 Nm/kg, Z = −2.8, *p* = 0.005 for full extension; −0.05 ± 0.08 Nm/kg, Z = −2.05, *p* = 0.04 for 135°; and −0.08 ± 0.09 Nm/kg Z = 2.95, *p* = 0.003 for 90°). The wrist moments presented left-right differences in the sagittal plane only in arm full extension condition (−0.03 ± 0.05 Nm/kg, Z = −2.39, *p* = 0.02). No differences were found for the elbow.

## 4. Discussion

The aim of the present study was to quantify muscle activities and joint loads during dead hangs performed with different arm lock-off positions. Our findings are in agreement with our hypothesis: the external joint moments in the shoulder and elbow increase with increasing elbow flexion in the arm lock-offs but muscle activations of the finger flexor muscles remained the same. These results highlight that different lock-off positions during dead hangs have the same training effect for finger flexor muscles but lead to different shoulder and elbow joint loads.

Increasing elbow flexion in the arm lock-offs resulted in higher elbow and shoulder moments. Although no previous studies have explored this specific isometric action, our findings are complementary to what has been reported for pull-ups. Variants of pull-ups involving different hand grip positions and orientations have been shown to significantly affect upper limb joint loads (25). It is known that the mechanical demands placed on the muscles and joints depend on the joint kinematics, and specific poses may increase pain and potentially the risk of pathology. For instance, rotator cuff related shoulder pain (RCRSP; historically called subacromial impingement syndrome), is a frequently reported shoulder condition in overhead athletes (26) as climbers (27). This condition has been formerly associated to glenohumeral instability as a primary cause (28), which would be facilitated by the smaller subacromial space at 120° of elevation, 90° of abduction and 45° of external rotation of the shoulder (29). However, recent literature has challenged the role of the impingement in the acromion in causing pathologies associated to pain in shoulder structures (30). Not only the recent tools are better capable of differentiating rotator cuff disorders (31), but it has been reported that exercise therapies presented the same benefits as acromioplasty, further putting impingement as the main symptom mechanism (32). The current consensus is that pain linked to poor mechanical load management in the performance of overhead activities are the most determining causal factors in RCRSP and its progression (33). Considering that dead hangs with arm lock-offs are commonly incorporated into training regimens to develop strength capacities, and most injuries in climbers occur due to overuse (7), it is crucial to prescribe them cautiously. Additionally, although study examined isometric exercises performed for a relatively short duration, it is known that exercising at intensities that induce fatigue and repetitive loading can alter muscle activations and joint kinematics and therefore the load distribution across upper extremity joints (34). The increased joint loads found for the shoulder in arm lock-offs can potentially represent a source RCRSP at long term.

We observed increased participation of the biceps brachii, trapezius, and latissimus dorsi with higher degrees of elbow flexion during arm lock-offs. These findings are consistent with studies on similar actions such as pull-ups and chin-ups, which have demonstrated that different angular positions in these movements elicit distinct recruitment strategies in the surrounding muscles (25). Furthermore, the greater involvement of the latissimus dorsi at 135° and 90° arm lock-offs compared to the biceps brachii and trapezius aligns with previous literature highlighting the latissimus dorsi as the most active muscle during these types of actions (25, 35, 36). Also, our results showed a high variability in the %RMS180° of the latissimus dorsi across participants. EMG is naturally affected by biological and instrumental sources of variability. Additionally, the shoulder is a joint with high degrees of freedom, therefore favoring variable length-tension outcomes, especially considering the large-volume of this muscle. However, the phenomena referenced as “climber's back” might also have a contribution to the variability of latissimus dorsi activations in the lock-off positions with increased elbow flexion. “Climber's back” is characterized by an imbalance between strong inwardly and weak outwardly muscles responsible to rotate the shoulder griddle, in combination with shortened pectoralis muscles (37). Although the present study did not monitor antagonist muscles, it is possible that participants might have had different levels of co-contraction and antagonist activity around the shoulder to maintain the lock-offs at high angles of elbow flexion, leading to the observed variability in the latissimus dorsi.

We found that left-right asymmetries in shoulder flexion and internal rotation moments tended to increase with increasing elbow lock-off angles. Functional asymmetries are inherent in symmetrical tasks performance (38, 39) but are also associated with increased risks for injuries (40). The objective of symmetry analysis in our study was not to emphasize the impact of side differences in performance, as this has been recently investigated in indoor climbing (41), but to comprehend the implications of potential asymmetries in shoulder and elbow moments during arm lock-offs. The findings of our study highlight that greater elbow flexion during isometric hangings may exacerbate the effects of sudden increased peak loads on the upper limb joints, thereby increasing the risk of injuries.

A worthy reflection to this discussion, which is critical to sports medicine and science, concerns the relationship between training load, injury, fitness, and performance. One might question: how can we help climbers enhance performance, knowing that repeated peak workloads result in pain and injuries and, at the same time, are necessary to elicit the adaptations that would make them stronger? The “Training-injury Prevention Paradox model”, by Tim Gabbett (42), debates over the fact that high training loads are necessary to enhance fitness and sport performance, but costs soft tissue injury risk. Moreover, lower workloads exposure is also related to susceptibility to injuries, thus training loads provide protective effect against it. The view about this dogma highlights the importance of monitoring load, so athletes are appropriately prescribed graded training loads to improve fitness and protect against pain and injury. In this sense, the primary purpose of dead hangs is typically to assess or improve finger flexor strength capacities (43–45), as hanging ability is a predictor of climbing performance (15, 46). Arm lock-offs are frequently incorporated into climbing-related tests (15), training protocols, and sport-specific movements (16, 47, 48). The present study provides novel and valuable insights into the functional aspects of isometric dead hangs with arm lock-offs, revealing the amount of load that climbers can expect to experience. The activation of the finger flexors remained unaffected by the increase in elbow flexion resulting from different lock-off angles, differently from the upper limb and trunk muscles, which increased participation. These findings would, then, support the recommendation to prescribe dead hang focusing on finger strength training with full elbows extension, thus minimizing unnecessary joint loading at elbows and shoulders. Still, it is reasonable that one might want to enhance strength capacities for back, shoulder, arm, and trunk muscles using climbing-oriented hand holds in overhead exercises. The optimization of this process needs to consider elements that would better translate to gains in sport performance and protect against pain and injury, thus pull-ups can be a better option to develop upper body strength and coordination in climbers (49).

Nevertheless, biomechanical modeling enables a comprehensive analysis of movements and loads applied to the musculoskeletal system (50). To the best of our knowledge, this study represents the first neuromechanical analysis of a specific exercises commonly used for strength training and assessments in climbing. The findings of this study have implications for training optimization in the sport. Coaches, trainers, and climbers can use this information as a guideline to develop smarter training protocols that target specific muscle groups and joint angles to enhance climbing performance while managing the factors related to upper limb joint pain and injuries.

## 5. Limitations and future directions

Our study includes the following limitations. First, we only evaluated muscle activity of a small set of muscles. We analyzed the primary muscles at the forearm, arm, and trunk that are used during the dead hangs with different arm lock-offs, which was sufficient to address our research questions. Additional investigations of antagonist muscles could provide insights into the stabilization strategies employed in the tasks. Second, our participants performed the dead hangs on one predefined hold size. Evaluating how joint moments and muscle activations change with varying hold sizes would enhance our understanding of the relationship between load distribution across the upper body joints and the increased involvement of finger flexor activity. Third, we only analyzed static, isometric dead hangs. Campus boarding, a common exercise in climbing, involves dynamic movement in combination with arm lock-offs, which might significantly increase joint loads. Hence, future studies should collect data from dynamic tasks to get a comprehensive overview of joint loads experienced during different climbing-specific movements. Fourth, we estimated finger strength training load solely based on the available EMG data, and no reliability measurement was performed. However, considering that we analyzed isometric exercises and the different lock-off positions did not alter the length of the forearm and finger muscles, we believe our estimations are reasonable and valid. Additionally, worth it mentioning that the vertical forces did not change across conditions, while lateral forces were slightly higher (in the order of 4 to 6 kg) in the lock-off positions. In the future, we plan to use musculoskeletal simulations to estimate in-vivo muscle forces during different climbing-related movements.

## 6. Conclusion

In summary, this study examined the neuromechanical characteristics of dead hangs with arm lock-offs at varying elbow flexion angles. The findings of this study offer valuable insights that can be applied to smarten training guidelines, once it demonstrates that performing isometric finger dead hangs with arms fully extended is an effective method for developing forearm force capacities. This exercise allows for targeted training of the forearm muscles while minimizing the strain on the elbow and shoulder joints. Overall, this study contributes to the understanding of the neuromechanical aspects of climbing-specific exercises, providing novel and applied information for climbers, trainers, and researchers in the field.

## Data Availability

The raw data supporting the conclusions of this article will be made available by the authors, without undue reservation and under request.
